# Histopathologically Confirmed Coronary Thromboangiitis Obliterans Successfully Treated With Surgical Revascularization

**DOI:** 10.1016/j.atssr.2025.08.019

**Published:** 2025-09-13

**Authors:** Joo yeon Kim, Young Tak Lee, Jeong-Wook Seo

**Affiliations:** 1 Department of Cardiovascular and Thoracic Surgery, Ewha Womans University Medical Center, Seoul, Republic of Korea; 2 Department of Cardiovascular and Thoracic Surgery, Incheon Sejong Hospital, Incheon, Republic of Korea; 3 Department of Pathology, Incheon Sejong Hospital, Incheon, Republic of Korea

## Abstract

Coronary and internal thoracic artery involvement in the absence of peripheral arterial disease is rare, and histopathologic confirmation is even more uncommon. A 52-year-old man presented with 3-vessel disease. Further evaluation studies suggested diffuse coronary periarteritis. Coronary artery bypass grafting was performed with corticosteroid therapy. During surgery, the internal thoracic artery also showed signs of vasculitis, with marked intimal thickening, rendering it unsuitable as a graft. Intraoperatively, the intima of the coronary arteries was thickened and easily dissected using a coronary blower, resembling coronary endarterectomy. Histopathologic examination confirmed thromboangiitis obliterans, also known as Buerger disease.

Thromboangiitis obliterans (TAO), commonly referred to as Buerger disease, is a rare, nonatherosclerotic inflammatory disease affecting small- and medium-sized arteries and veins, most commonly in the extremities of young male smokers. TAO is typically diagnosed clinically based on exclusion of other vascular disease. Because the diagnosis is primarily based on clinical criteria,^1^ pathologic confirmation is uncommon.^2^ Furthermore, coronary involvement is extremely rare, and surgical management remains challenging.^3^ In this report, we describe a rare case of coronary and internal thoracic artery (ITA) involvement confirmed by histopathology in a patient without peripheral vascular symptoms, who underwent surgical revascularization.

A 52-year-old man with old smoking history, hypertension, and dyslipidemia initially presented with persistent chest discomfort. He was diagnosed with unstable angina with 3-vessel disease, including chronic total occlusion of the left anterior descending artery and diffuse stenosis of the right coronary artery at another hospital in July 2023 (Figure 1). He initially received medical treatment but was referred to Incheon Sejong Hospital for further management because his symptoms continued to worsen despite initial conservative treatment.

**Figure 1:**
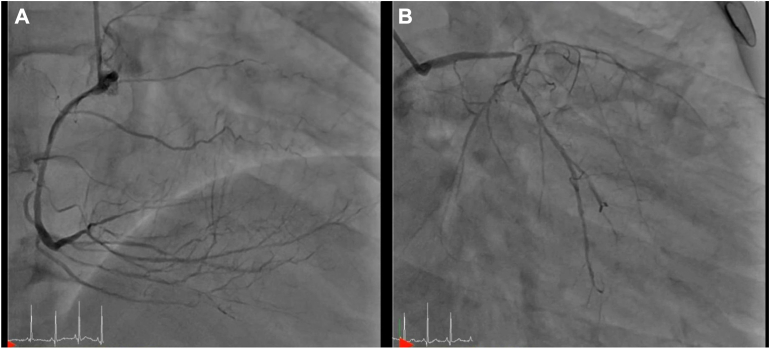
The coronary angiography showed diffuse stenosis of right coronary artery (A) and left circumflex artery with chronic total occlusion of the left anterior descending artery (B).

A repeated coronary angiography and heart computed tomography revealed diffuse coronary artery disease with severe stenosis, raising suspicion for an underlying vasculitic process rather than atherosclerotic disease. Initially, serum immunoglobulin G subclass IV was elevated. Given the clinical findings, the possibility of immunoglobulin G–related vasculitis was considered, and high-dose corticosteroid therapy was initiated. The patient received prednisolone at 60 mg/d for 12 days, which was subsequently tapered and discontinued by 2 months after surgery. A repeat coronary angiography 2 weeks later showed mild improvement in stenosis, but significant residual disease remained, prompting the decision to proceed with surgical revascularization.

After 4 weeks of corticosteroid therapy, the patient underwent conventional coronary artery bypass grafting under cardioplegic arrest. During the procedure, both ITAs were found to be extensively involved with vasculitis, with marked intimal thickening and luminal narrowing. Based on their gross appearance, they were deemed unsuitable for use as grafts. Instead, a saphenous vein graft (SVG) was harvested for revascularization. The first segment of the SVG was anastomosed from the ascending aorta to the left anterior descending, and the other SVG segment was grafted from the aorta to the right intermediate, posterolateral, and posterior descending artery branches sequentially.

Notably, the coronary arteries were found to have thickened walls, and the intima was easily dissectible using a blower, indicating significant inflammatory involvement. Before endarterectomy, the luminal diameter was severely narrowed to the extent that it could not be reliably measured. Given the nature of the disease, larger-than-usual arteriotomies were required to facilitate graft anastomosis, and endarterectomy-like removal of the diseased intima was necessary to ensure optimal blood flow through the grafts (Figure 2).

**Figure 2:**
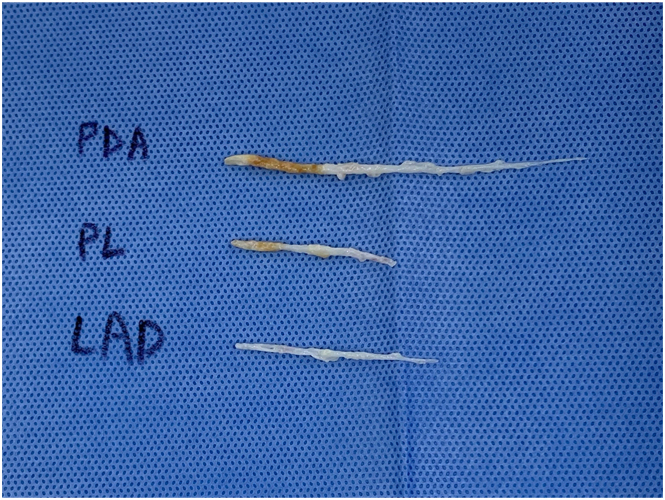
The intima of coronary arteries was easily dissectible using a blower. We performed endarterectomy-like removal of the diseased intima. (LAD, left anterior descending; PDA, posterior descending artery; PL, posterolateral branch.)

Histopathologic examination of the ITA excised intima confirmed features consistent with TAO, including marked intimal hyperplasia and inflammatory infiltration (Figure 3A). The longitudinal section of the coronary arterial plug that was from Figure 3 also showed same features of internal elastic lamina (Figure 3B). The vessel walls displayed a dense perivascular lymphocytic infiltration with no evidence of atherosclerosis, further supporting the diagnosis of vasculitis rather than traditional coronary artery disease.

**Figure 3:**
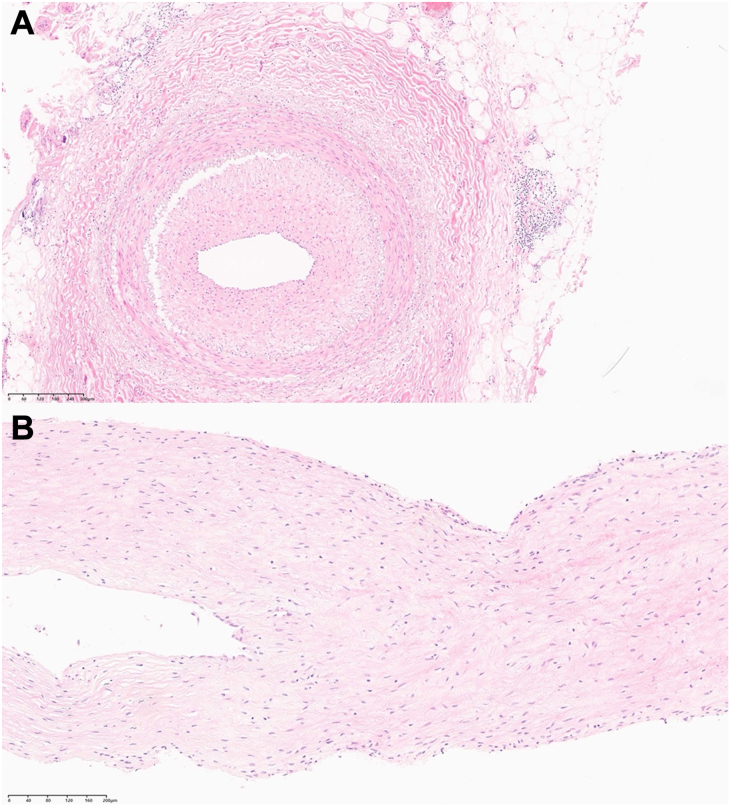
(A) Histopathologic examination showed marked thickening of the intimal layer and disruption of the intima from the internal elastic lamina. (B) Longitudinal section of the coronary arterial plug also showed same features of intimal hyperplasia of internal elastic lamina. Scale bar = 200 μm.

The patient was successfully weaned off cardiopulmonary bypass without complications. Other postoperative treatments were performed according to routine management. By postoperative day 14, he had recovered sufficiently to be discharged home in stable condition. His discharge medications included edoxaban, 30 mg daily, and Plavix (Clopidogrel), 75 mg daily, with continued corticosteroid tapering. At his 12-month follow-up, the patient remained asymptomatic with no recurrent ischemic events. Repeat imaging confirmed patent grafts and stable cardiac function. He was advised to continue aggressive risk factor modification, including smoking cessation and regular cardiovascular monitoring.

## Comment

TAO is not a life-threatening disease, but it is one of the debilitating peripheral vascular diseases that can lead to limb amputation, significantly impacting a patient’s quality of life.^1^ Although diagnostic criteria, such as the Shionoya Classification exist, recent studies in Japan have shown that only 12% of patients meet these criteria,^4^ highlighting the increasing difficulty in diagnosing TAO.

Histopathologic confirmation is not required for the diagnosis of TAO and is rarely performed. In chronic or “old” lesions, the literature often describes the presence of organized thrombi^2^; however, such findings were not observed in this patient. Instead, the diagnosis was supported by reduplication and hyperplasia of the internal elastic lamina,^5^ along with evidence of disruption, consistent with vasculitic changes.

Although TAO is commonly suspected in young patients aged <50 years, diagnosis and treatment become more challenging in elderly individuals due to the frequent overlap with atherosclerosis.^6^ Furthermore, coronary involvement is a rare complication of TAO, and cases in which coronary artery disease occurs without peripheral vascular involvement are even rarer, making diagnosis in such instances exceptionally difficult. Through this case study, TAO may be suspected in the presence of diffuse coronary disease in a young patient with a history of smoking.

No gold standard treatment has been established for TAO. However, as demonstrated in this case, a combination of vasculitis-targeted therapy and revascularization techniques,^7^ including an endarterectomy-like approach, may be a viable therapeutic strategy for selected patients.

Importantly, the perioperative use of corticosteroids—often required for systemic vasculitis—has been a subject of concern due to potential risks of impaired wound healing and infection. However, a propensity-matched analysis by Pai and colleagues^8^ demonstrated that chronic steroid therapy before cardiac surgery was not associated with increased operative mortality or major morbidity, suggesting that surgical intervention may still be safely performed in appropriately selected patients receiving long-term corticosteroids.

This case highlights the importance of considering surgical revascularization in a patient with vasculitic coronary disease when medical therapy alone is insufficient.
